# Characteristics of people with bipolar disorder I with and without auditory verbal hallucinations

**DOI:** 10.1186/s40345-025-00369-8

**Published:** 2025-02-14

**Authors:** Aster Javier, Natalia Jaworska, Jess Fiedorowicz, Vincent Magnotta, Jenny G. Richards, Ercole John Barsotti, John A. Wemmie

**Affiliations:** 1 University of Ottawa Institute of Mental Health Research, Ottawa, Canada; 2 Department of Neuroscience, Carleton University, Ottawa, Canada; 3 Cellular & Molecular Medicine, University of Ottawa, Ottawa, Canada; 4 Ottawa Hospital Research Institute, University of Ottawa, Ottawa, Canada; 5 Department of Radiology, University of Iowa, Iowa, USA; 6 Department of Psychiatry, University of Iowa, Ottawa, USA; 7 1145 Carling Avenue, Room 3129, Ottawa, ON K17 7K4 Canada

**Keywords:** Bipolar disorder, Auditory verbal hallucinations, Psychotic features, Sample characteristics

## Abstract

**Background:**

Approximately half of people with bipolar disorder type I (BD-I) report the presence of psychotic symptoms at least at some point during their illness. Previous data suggest that more than 20% of people with BD-I report the presence of auditory verbal hallucinations (AVHs), or “voice-hearing” in particular. While work in other disorders with psychotic features (e.g., schizophrenia) indicates that the presence vs. absence of AVHs is associated with poorer clinical outcomes, little is known about their effects on clinical and socioeconomic features in BD-I.

**Methods:**

We investigated whether people with BD-I (*N* = 119) with AVHs (*n* = 36) and without AVHs (*n* = 83) in their lifetime differ in terms of demographic features and clinical measures. Relations with AVHs and other positive symptoms were explored.

**Results:**

People with BD-I and AVHs vs. without AVHs had higher manic and positive symptom scores (i.e., higher scores on the hallucinations, delusions, and bizarre behavior subscales). Further, a greater proportion of those with vs. without AVHs reported lower subjective socioeconomic status and tended to have higher rates of unemployment, thus, speaking to the longer-term consequences of AVH presence.

**Conclusion:**

Our findings suggest that people with BD-I with AVHs exhibit more severe psychotic features and manic symptoms compared to those without. This might be associated with more socioeconomic hardship. More in-depth characterization of people with BD-I with/without AVHs is needed to fully understand this subgroup’s unique challenges and needs.

**Limitations:**

The modest sample size of the AVH group and a study population with low racial diversity/representation may limit generalizability.

**Supplementary Information:**

The online version contains supplementary material available at 10.1186/s40345-025-00369-8.

## Background

Bipolar disorder type I (BD-I) is a debilitating mood disorder characterized by episodes of mania (i.e., periods of abnormally elevated and irritable mood) and depression. However, psychotic symptoms, such as hallucinations and delusions, can also occur in BD-I, with at least one lifetime occurrence being reported in up to 57–70% of people with BD-I (Aminoff et al. 2022; Upthegrove et al. 2015). Studies have shown that a history of psychotic symptoms in BD-I is associated with increased cognitive and functional impairment, as well as a greater number of hospitalizations (Glahn et al. 2007; Levy et al. 2013; Simonsen et al. 2011; Strakowski et al. 2000).

Though generally understudied in the context of BD, previous work indicates that approximately 20% of people with BD-I report experiencing auditory verbal hallucinations (AVH), one of the most common types of hallucinations, at least once during their illness course (Morgan et al. 2005; Toh et al. 2015). AVHs can occur in any state of BD-I, but have been found to be more present during manic and mixed-manic episodes (Smith et al. 2017). Work in other disorders with psychotic features, such as schizophrenia, indicates that AVHs are associated with poorer clinical outcomes, as well as greater cognitive and functional impairments (Hugdahl et al. 2013; Jenkins et al. 2018; Kelleher et al. 2013; Toh et al. 2015). Studies in people with schizophrenia have also reported that AVHs correlated with increased severity of other hallucination types; this might contribute to functional impairment (Eve Lewandowski et al. 2009; Mueser et al. 1990). AVHs in people with BD-I have been associated with reduced illness insigh; that is, decreased awareness of symptoms, openness to feedback about illness, and self-reflectiveness (Baethge et al., 2005; Kumari et al. 2013). Decreased insight has been linked with medication non-adherence, reduced cognitive performance, and daily and occupational functioning impairments (Sajatovic et al. 2011; Wels et al. 2023). Conversely, increased illness insight has been linked with better coping and adaptation in BD-I patients (Tohumcu and Çuhadar 2024). Compared to people with schizophrenia, the impact of AVHs in the context of BD-I in terms of sociodemographic features and clinical profiles has been unexplored. Such insight might be important in laying the groundwork for more tailored and ultimately more effective treatment approaches for distinct subgroups of people with BD-I.

This study aimed to assess whether BD-I patients with vs. without a history of AVH differ in terms of demographic (i.e., age, assigned sex at birth, race, ethnicity) and clinical features (e.g., depressive, manic, and positive symptoms). Most novel, we also assessed occupational and daily functioning, operationalized by years of education, subjective socioeconomic status (SES), employment/disability/student status, and housing situation to assess the influence of AVH presence in people with BD. We expected that patients with AVHs would exhibit more pronounced indications of functional impairments, such as increased unemployment and disability status, and lower levels of education and subjective SES. We also explored if AVH severity was related to the severity of other hallucination types in people with BD-I and AVHs. Though this has been studied in schizophrenia, positive symptoms do not present the same way in BD-I (Baethge et al., 2005; Mancuso et al. 2015), as such, exploring such relations in people with BD-I addresses this research gap.

## Methods

### Participants

A total of *N* = 119 people with BD-I, aged 18–68 years (39.3, S.D.=13.5), were recruited as part of the Bipolar Disorder Research Program of Excellence at the University of Iowa; participants were either outpatients affiliated with the University Health Centre or recruited from the community. To ensure eligibility and confirm diagnosis, research personnel administered the Structured Clinical Interview for DSM-5 (SCID-5) (First et al. 2015), followed by an unstructured clinical interview by a psychiatrist. The majority (64.7%; *N* = 77) were female sex assigned at birth. Participants were divided into two groups based on the first item of the Scale for the Assessment of Positive Symptoms (SAPS) (Andreasen 1984): those who experienced AVH in their lifetime (BP/AVH+, SAPS score ≥ 1; *N* = 36) and those who did not (BP/AVH-, SAPS score = 0; *N* = 83). Group (i.e., BP/AVH + vs. BP/AVH-) features are presented in Table 1.

### Measures

The SAPS is a 34-item scale that measures positive psychotic symptoms (Andreasen 1984). It is divided into 4 subscales measuring hallucinations (items 1–6), delusions (8–19), bizarre behavior (21–24), and positive formal thought disorder (26–33). Each item is rated from 0 (absent) to 5 (severe). There are also global items (self-reported overall ratings for each subscale), but these were not included in the total/composite SAPS score or any subscale totals. Reliability tests showed excellent internal consistency of the overall SAPS scale (Cronbach’s alpha = 0.91) in our sample (*N* = 119).

The Montgomery Åsberg Depression Rating Scale (MADRS) is a 10-item scale that measures the presence of depression symptoms in the last week (Davidson et al. 1986). Each item is rated from 0 (none) to 6 (severe). Reliability tests showed good internal consistency (Cronbach’s alpha = 0.83) in our sample (*N* = 119).

The Young Mania Rating Scale (YMRS) is an 11-item scale that measures the presence of manic symptoms in the past 48 h (Young et al. 1978). Items 1–5, 7, 10, 11 are rated from 0 (none) to 4 (severe), while items 6, 8, 9 are rated 0 (none) to 8 (severe). Reliability tests showed acceptable internal consistency (Cronbach’s alpha = 0.78) in our sample (*N* = 119).

Subjective SES was obtained using a “ladder rung” item that asked participants to select where they believe they rank socioeconomically (Adler and Stewart 2007). The bottom rung corresponds to a score of 1 (lowest SES), while the highest rung corresponds to a score of 10 (highest SES).

Retrospective symptom burden for each participant was estimated by percentage of time (self-reported) spent in depressed, manic, and euthymic mood states over the past decade (maximum 100%).

### Statistics

Statistical analyses were performed using SPSS for Mac v. 28.0 (SPSS, Chicago, Illinois, USA); significance levels were *p =* 0.05, unless stated otherwise. A two-tailed student t-test was run to compare groups (i.e., BP/AVH + vs. BP/AVH-) on age. One-sided t-tests were applied for total years of education and subjective SES as the AVH group was expected to report lower levels. Two-sided Pearson Chi Square statistics were used to compare groups on housing (homeless/shelter, living with family, owns a house, or renting an apartment/dormitory), assigned sex at birth, race, and ethnicity. To further explore our directional hypothesis (AVH group would exhibit more functional impairments), a Fisher’s exact test (one-sided) was used to compare groups on disability status (persons with or without disabilities), employment (full/part time employment or unemployed), and student status (enrolled in school or not).

Separate one-way analyses of variance (ANOVAs) were used to compare groups on SAPS, MADRS, and YMRS total scores. A multivariate ANOVA (MANOVA) was also used to compare groups on the four SAPS subscales. For the AVH group, exploratory Pearson’s correlations (with bias-corrected and accelerated bootstrapping) were performed to examine relations between the SAPS AVH item and each of the following SAPS items: tactile/somatosensory hallucinations, visual, and olfactory hallucinations. Finally, Mann-Whitney U tests were performed to compare the two groups on the self-reported and retrospectively estimated percentage of time spent in depressed, manic, and euthymic mood states over the past decade.

## Results

### Demographics

Table 1 displays the means for various demographic factors in the BP/AVH + and BP/AVH- groups. The groups did not differ in terms of age (t(1,117) = 0.66, *p =* 0.51), assigned sex at birth (χ^2^ = 0.92, *p =* 0.34), race (χ^2^ = 1.81, *p =* 0.77), ethnicity (χ^2^ = 4.71, *p =* 0.09), or housing situation (χ^2^ = 0.85, *p =* 0.84). The AVH group tended to have fewer years of education than the group without AVHs (t(1,117)=-1.35, *p =* 0.09). The BP/AVH + group had a lower subjective SES (t(1,116)=-1.58, *p =* 0.05) and a greater tendency for being unemployed (χ^2^ = 2.87, *p =* 0.068) compared to the BP/AVH- group. Fisher’s exact tests revealed that the AVH group was not more likely to declare disability status (χ^2^ = 0.76, *p =* 0.26) or be out of school (χ^2^ = 0.43, *p =* 0.37) compared to the group without AVHs; the inclusion of total SAPS scores (minus hallucinatory item score) as a covariate on these outcomes is presented in Supplementary Table 1.

**Table 1:** Demographic factors and clinical scores for people with bipolar disorder type I (BD-I) with (BP/AVH+) and without (BP/AVH-) auditory verbal hallucinations (AVH)

Parameter	BP/AVH+ (*N* = 36)	BP/AVH- (*N* = 83)	Significance (*N* = 119)
Age (Years)	M = 40.6 (SD = 13.5)	M = 38.8 (SD = 13.9)	t(1,117) = 0.66 *p =* 0.51
Total Years of Education	M = 14.5 (SD = 2.0)	M = 15.0 (SD = 2.2)	t(1,117)=-1.23 1-tailed *p =* 0.11
Subjective SES	M = 4.8 (SD = 2.0)	M = 5.4 (1.9)	t(1,116)=-1.58 1-tailed *p =* 0.05*
Assigned Sex at Birth	Female = 21 (58.4%) Male = 15 (41.6%)	Female = 56 (67.5%) Male = 27 (32.5%)	χ^2^ = 0.92 *p =* 0.34
Race	Native American = 0 (0%) Asian = 0 (0%) Black/African American = 2 (5.6%) Multiracial = 1 (2.7%) White = 33 (91.7%)	Native American = 2 (2.4%) Asian = 2 (2.4%) Black/African American = 4 (4.8%) Multiracial = 2 (2.4%) White = 73 (88.0%)	χ^2^ = 1.81 *p =* 0.77
Ethnicity	Hispanic/Latino = 3 (8.3%) Other Ethnicity = 31 (86.1%) Unknown = 2 (5.6%)	Hispanic/Latino = 8 (9.6%) Other Ethnicity = 75 (90.4%) Unknown = 0 (0%)	χ^2^ = 4.71 *p =* 0.09
Housing Situation	Homeless/shelter = 1 (2.8%) Living with family = 5 (13.9%) House owner = 14 (38.9%) Renting = 16 (44.4%)	Homeless/shelter = 1 (1.2%) Living with family = 15 (18.1%) House owner = 28 (33.7%) Renting = 39 (47.0%)	χ^2^ = 0.85 *p =* 0.84
Disability Status	Disability = 10 (27.8%) Without disability = 26 (72.2%)	Disability = 17 (20.5%) Without disability = 66 (79.5%)	χ^2^ = 0.76 *p =* 0.26 (1-sided)
Employment Status	Employed (full/part time) = 17 (47.2%) Unemployed = 19 (52.8%)	Employed (full/part time) = 53 (63.9%) Unemployed = 30 (36.1%)	χ^2^ = 2.87 *p =* 0.068 (1-sided)
Student Status (full/part time)	Student = 4 (11.1%) Not in school = 32 (88.9%)	Student = 13 (15.7%) Not in school = 70 (84.3%)	χ^2^ = 0.43 *p =* 0.37 (1-sided)
SAPS total	M = 25.3 (SD = 16.0)	M = 5.2 (SD = 6.2)	*F*(1,117) = 98.28 *p <* 0.001**
MADRS total	M = 14.8 (SD = 7.9)	M = 14.0 (SD = 9.7)	*F*(1,117) = 0.16 *p =* 0.69
YMRS total	M = 10.1 (SD = 8.6)	M = 5.2 (SD = 5.5)	*F*(1,117) = 13.78 *p* < 0.001**
% time (past decade): Depressed	M = 38.8 (SD = 22.9)	M = 38.4 (SD = 26.2)	Z=-0.20 *p =* 0.84
% time (past decade): Manic	M = 23.0 (SD = 16.9)	M = 16.8 (SD = 16.0)	Z=-2.13 *p =* 0.03*
% time (past decade): Euthymic	M = 38.2 (SD = 26.7)	M = 44.9 (SD = 29.8)	Z=-1.01 *p =* 0.31

*p ≦ 0.05, **p < 0.001. Note. SES: Socioeconomic Status; SAPS: Scale for the Assessment of Positive Symptoms; MADRS: Montgomery Åsberg Depression Rating Scale; YMRS: Young Mania Rating Scale

### Clinical measures

In terms of clinical scales (Table 1), SAPS (F(1,117) = 98.28, *p <* 0.001, _partial_η^2^ = 0.46) and YMRS (F(1,117) = 13.78, *p <* 0.001, _partial_η^2^ = 0.11) total scores were elevated in the BP/AVH + vs. BP/AVH- group. However, AVH presence was not found to influence MADRS total scores (F(1,117) = 0.16, *p =* 0.69, _partial_η^2^ = 0.001; see Supplementary Table 1 for the effect of including total SAPS score [minus hallucinatory item] as a covariate).

**Fig. 1:**
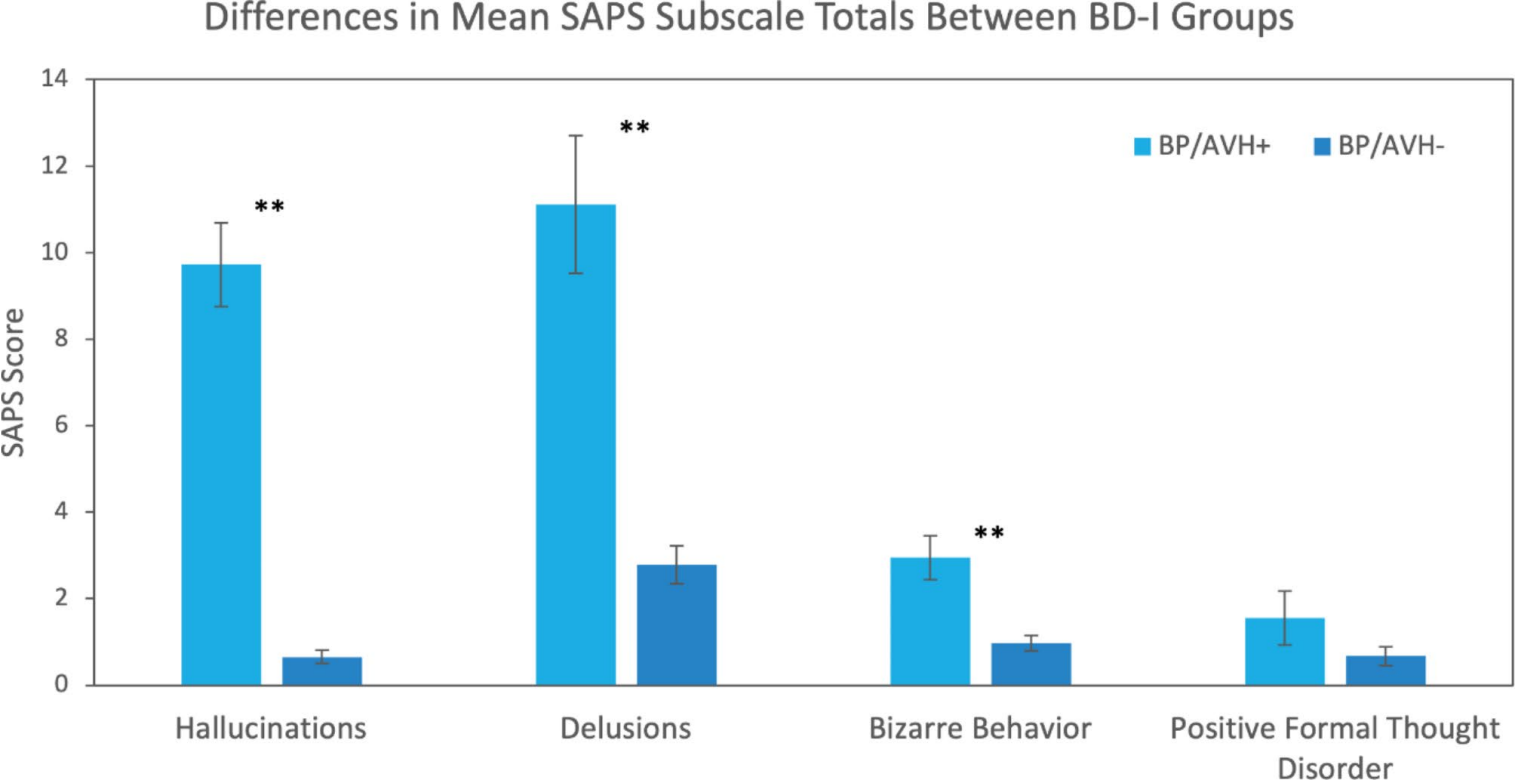
Differences Between People with Bipolar Disorder Type I (BD-I) with (BP/AVH+) and without (BP/AVH-) Auditory Verbal Hallucinations (AVH) on the Scale for the Assessment of Positive Symptoms (SAPS) Subscales: Hallucinations, Delusions, Bizarre Behavior, and Positive Formal Thought Disorder. ** *p <* 0.001

A MANOVA for the four SAPS subscales (hallucinations, delusions, bizarre behavior, positive formal thought disorder) was conducted between the groups; the multivariate model was significant (Pillai’s Trace = 0.61, F(4,114) = 47.97, *p <* 0.001, _partial_η^2^ = 0.63). Follow-up univariate analyses yielded a difference between groups on hallucinations (F(1,117) = 192.44, *p <* 0.001, _partial_η^2^ = 0.62), delusions (F(1,117) = 43.92, *p <* 0.001, _partial_η^2^ = 0.27), and bizarre behavior (F(1,117) = 17.64, *p <* 0.001, _partial_η^2^ = 0.13) subscales (Fig. 1). No group difference existed on the positive formal thought disorder subscale (F(1,117) = 2.10, *p =* 0.15, _partial_η^2^ = 0.02).

Finally, the BP/AVH + group (M = 23.0%) reported significantly more time spent in manic episodes over the past decade compared to the BP/AVH- group (M = 16.8%) (Z=-2.13, *p =* 0.03). These differences were not evident for depressed (Z=-0.20, *p =* 0.84) or euthymic (Z=-1.01, *p =* 0.31) states.

### Exploratory correlations

For the BP/AVH + group, there was no significant correlations found between the SAPS AVH and tactile hallucination items (*r* = 0.004, *p =* 0.63, 95% BCa CI [-0.40, 0.35]). This was also the case for the correlation between the AVH and the olfactory hallucination (*r* = 0.07, *p =* 0.67, 95% BCa CI [-0.26, 0.39]) and visual hallucination items (*r* = 0.18, *p =* 0.28, 95% BCa CI [-0.15, 0.48]).

## Discussion

This study aimed to compare demographic and clinical characteristics between BD-I people with vs. without AVHs. We expected that the AVH group would exhibit more functional impairments in terms of socioeconomic (e.g., unemployment, disability status) and clinical features. In addition, we sought to examine the relations between the SAPS AVH item and other types of hallucinations for the BP/AVH + group as this has previously been under-explored and would provide some insight into the relations between hallucinatory features.

Our BP/AVH + sample made up 30.3% (*n* = 36) of the entire cohort (*N* = 119), which is consistent (though somewhat higher) with past literature that found the proportion of people with BD-I and AVHs was around 20% (Morgan et al. 2005; Toh et al. 2015). While we found that the groups did not differ on most demographic factors, people with BD-I and AVHs reported lower subjective SES and a tendency for lower employment rates compared to those without AVHs. The AVH group also had elevated positive (delusions, bizarre behavior, other kinds of hallucinations) and manic symptoms; no differences in depressive symptoms existed. In addition, the AVH group had higher YMRS scores and retrospectively reported significantly more time spent in manic states over the past decade compared to the non-AVH group. Finally, no correlations were found between the SAPS AVH item and any of the hallucination items for the BP/AVH + group.

People with BD-I and AVHs were found to have lower subjective SES and a tendency for lower employment rates than those without AVHs, which aligns with our hypotheses. However, while we expected the AVH group to exhibit functional impairments as assessed by years of education, disability status, housing situation, and student status, there was no significant difference found between groups. Previous research on people with AVHs (general population and those with schizophrenia only) found several indications of functional impairment in addition to lower SES and unemployment (Kr Akvik et al. 2015; Varese et al. 2011). Others have noted that people with BD and schizophrenia with AVHs vs. without AVHs exhibited greater working memory impairments (Gupta et al. 2018; Jenkins et al. 2018), and abnormalities in neural networks related to attention, emotion processing, speech production/processing, and memory (Kühn and Gallinat 2012; Qiu et al. 2020). By extension, such cognitive and emotional impairments could translate to daily functional impairments. A meta-analysis (Depp et al. 2012) reported significant relations between cognitive abilities (i.e., verbal learning, working and visual learning memory) and daily functioning (measured by clinician and self-report ratings, functional milestones, and performance-based tasks) in BD-I patients. Studies have also reported correlations between cognitive function and social and occupational functioning in both symptomatic and euthymic BD-I patients (Baune and Malhi 2015). Our data suggest functional impairments in people with BD-I and AVHs as operationalized by lower self-reported SES and a tendency for decreased employment rates, speaking perhaps to lower socioeconomic productivity in this cohort. Granted, other positive symptoms/greater illness severity likely accounted for some of this variance (see Supplementary Table 1); larger epidemiological studies are required to home in on the specific contributions of AVHs on clinical and socioeconomic features of this feature in BD.

The AVH group exhibited increased positive symptoms – specifically, hallucinations (AVHs as well as other types), delusions, and bizarre behavior – in comparison to the non-AVH group. These results align with previous research in people with schizophrenia (Chang et al. 2015; Chen et al. 2015; Larøi et al. 2012; Wang et al. 2021) and BD (Smith et al. 2017; Toh et al. 2015). Collectively, this suggests that the presence of AVHs corresponds to the worsening of other psychotic symptoms. Past studies have shown that positive symptoms are correlated with each other; further, greater positive symptoms (especially hallucinations and delusions) predicted higher rates of illness recurrence, relapse, hospital admissions, and suicide attempts in BD (Smith et al. 2017; Wilson and Sponheim 2014), underscoring the need for more proactive and intensive treatment approaches for this population. The only positive symptom that the groups did not significantly differ on was positive formal thought disorder. Past studies have shown that this symptom is more severe in people with schizophrenia compared to people with BD-I (Yalincetin et al. 2017), which could explain our results.

AVH presence was accompanied by elevated manic symptoms, as well as more time spent in manic episodes over the past decade (retrospective, self-report). Several studies have also reported that AVHs were more associated with manic and mixed-manic episodes in people with BD (Baethge et al. 2005b; Smith et al. 2017). This may indicate a connection between the co-occurrence of mania and psychotic features in BD-I. Indeed, significant overlap has been observed between manic and positive symptoms, which corroborates the proposition of psychosis as a spectrum disorder that includes BD-I (Craddock et al. 2009; Dunayevich et al. 2000; Simonsen et al. 2011). These findings suggest that people with BD-I and AVHs (as well as other positive symptoms) experience exacerbated, more chronic manic symptoms, which can lead to lower quality of life and poorer clinical outcomes. This highlights the need for specialized early detection and treatment strategies that address psychosis and mania in concert.

No differences existed between groups on depressive symptom severity. Psychotic features in BD, such as AVHs, can manifest during manic and depressive (and mixed) episodes (Chakrabarti and Singh 2022). Some have reported that AVHs in people with BD-I are less associated with depressive vs. manic episodes, while others report the opposite (Hammersley et al. 2010; Smith et al. 2017; Toh et al. 2015). AVH severity in patients with schizophrenia was associated with more severe depressive symptoms and lower social functioning (Abdelraof et al. 2023; Wang et al. 2019). It is feasible that AVHs have less influence/interaction with depression features and depression severity in BD-I; however, this needs to be assessed with larger samples.

Finally, our exploratory analyses indicated that there were no significant correlations between AVH severity and the severity of other hallucination types for the BP/AVH + group. While there is limited precedent literature on this, our findings are inconsistent with previous work noting correlations between AVHs and other types of hallucinations (visual, tactile/somatosensory, and olfactory) in people with schizophrenia (Eve Lewandowski et al. 2009; Mueser et al. 1990). It is feasible that the relatively modest severity of AVHs in our sample might contribute to the lack of notable relations with other hallucinatory features.

While novel, several limitations of the above work must be acknowledged. First, only a small percentage (~ 11%; *n* = 13) of the cohort identified as a racial minority, which may impact generalizability. Second, the smaller sample of people with BD-I and AVHs (vs. without) may result in less precise power estimates. Future studies with larger and more diverse samples are warranted to fully investigate the clinical and socioeconomic effects of AVHs in people with BD-I. Further, the retrospective symptom burden data was collected using self-report methods rather than direct measurements; these might be impacted by both recall bias and insight into symptoms, and limits reliability. While only used to supplement findings from clinical rating analyses, we acknowledge this method has not been validated. Nevertheless, the retrospective data on mania corroborate and expand upon the YMRS symptom data by offering a glimpse into long-term manic profiles. Also, we acknowledge that the use of subjective SES might be a limitation. Income was not collected as part of the study, but this measure might not have accurately captured SES in our cohort. This study was conducted in the metro area of Iowa, a university/college town with ~ 30,000–40,000 students (out of a population of ~ 75,000). These students might report a low income because of part-time employment or no employment during school; thus, years of education might better reflect their objective SES. Nevertheless, objective measures of SES should be utilized in future studies.

Finally, after completing data collection and analyses, it was discovered that portion of people with BD and AVH- (*N* = 30) were asked about their hallucinatory status in the *past month* (vs. lifetime, as was asked for the remainder of participants [*N* = 53]). As such, it is feasible that these people were miscoded (i.e., a portion of the *N* = 30 might have been lifetime hallucinators). To address this possibility, sensitivity analyses were carried out by excluding these *N* = 30 people from our AVH- group. Our results did not change substantially; the exception was that disability status was no longer significant while SES reached significance (likely due to power issues). This sensitivity analyses, coupled with the fact that the inclusion of the *N* = 30 people with BD and AVH- in the past month (vs. lifetime) biases analyses to the null hypotheses, and that -based on previous work- only a minority would be miscoded, suggests that our data, as presented, are robust.

In summary, AVHs in people with BD-I were found to be associated with functional impairments manifesting as unemployment and subjective socioeconomic hardship, as well as more severe positive (specifically other types of hallucinations, delusions, and bizarre behavior) and manic symptoms. While this research contributes to the growing body of research characterizing people with BD-I and AVHs, larger-scale studies with more in-depth investigation into demographic, socioeconomic, cognitive, clinical, and neural features are needed. Such insight might inform more tailored treatment approaches for this clinical population, thus improving functional outcomes and quality of life.

## Electronic supplementary material

Below is the link to the electronic supplementary material.

Supplementary Material 1

## Data Availability

Due to the sensitive nature of the questions asked in this study, survey respondents were assured raw data would remain confidential and would not be shared.

## Declarations

### Data statement

Due to the sensitive nature of the questions asked in this study, survey respondents were assured raw data would remain confidential and would not be shared..

## References

[CR1] Abdelraof A, Hamed S, Abd ELhay E. The impact of auditory hallucinations severity on social skills and depressive symptoms among schizophrenic patients. Mansoura Nurs J. 2023;10(1):155–66. 10.21608/MNJ.2023.320383.

[CR2] Adler NE, Stewart J. (2007). The MacArthur Scale of Subjective Social Status. *MacArthur Research Network on SES and Health at UCSF*.

[CR3] Aminoff SR, Onyeka IN, Ødegaard M, Simonsen C, Lagerberg TV, Andreassen OA, Romm KL, Melle I. (2022). Lifetime and point prevalence of psychotic symptoms in adults with bipolar disorders: a systematic review and meta-analysis. In *Psychological Medicine* (Vol. 52, Issue 13). 10.1017/S003329172200201X 10.1017/S003329172200201X PMC9647517 36016504

[CR4] Andreasen NC. (1984). Scale for the Assessment of positive symptoms (SAPS). Br J Psychiatry Suppl, *0*(7).

[CR5] Baethge C, Baldessarini RJ, Freudenthal K, Streeruwitz A, Bauer M, Bschor T. Hallucinations in bipolar disorder: characteristics and comparison to unipolar depression and schizophrenia. Bipolar Disord. 2005a;7(2):136–45. 10.1111/J.1399-5618.2004.00175.X. 15762854 10.1111/j.1399-5618.2004.00175.x

[CR6] Baethge C, Baldessarini RJ, Freudenthal K, Streeruwitz A, Bauer M, Bschor T. Hallucinations in bipolar disorder: characteristics and comparison to unipolar depression and schizophrenia. Bipolar Disord. 2005b;7(2):136–45. 10.1111/J.1399-5618.2004.00175.X. 15762854 10.1111/j.1399-5618.2004.00175.x

[CR7] Baune BT, Malhi GS. A review on the impact of cognitive dysfunction on social, occupational, and general functional outcomes in bipolar disorder. Bipolar Disord. 2015;17:41–55. 10.1111/BDI.12341. 26688289 10.1111/bdi.12341

[CR8] Chakrabarti S, Singh N. Psychotic symptoms in bipolar disorder and their impact on the illness: a systematic review. World J Psychiatry. 2022;12(9):1204. 10.5498/WJP.V12.I9.1204. 36186500 10.5498/wjp.v12.i9.1204 PMC9521535

[CR9] Chang X, Xi Y-B, Cui L-B, Wang H-N, Sun J-B, Zhu Y-Q, Huang P, Collin G, Liu K, Xi M, Tan Q-R, Miao D-M, Yin H. (2015). *Distinct inter-hemispheric dysconnectivity in schizophrenia patients with and without auditory verbal hallucinations OPEN*. 10.1038/srep11218 10.1038/srep11218 PMC4459220 26053998

[CR10] Chen X, Liang S, Pu W, Song Y, Mwansisya TE, Yang Q, Liu H, Liu Z, Shan B, Xue Z. Reduced cortical thickness in right Heschl’s gyrus associated with auditory verbal hallucinations severity in first-episode schizophrenia. BMC Psychiatry. 2015;15(1):1–8. 10.1186/S12888-015-0546-2/FIGURES/1. 26149490 10.1186/s12888-015-0546-2 PMC4493802

[CR11] Craddock N, O’Donovan MC, Owen MJ. Psychosis Genetics: modeling the relationship between Schizophrenia, bipolar disorder, and mixed (or Schizoaffective) psychoses. Schizophr Bull. 2009;35(3):482–90. 10.1093/SCHBUL/SBP020. 19329560 10.1093/schbul/sbp020 PMC2669589

[CR12] Davidson J, Turnbull CD, Strickland R, Miller R, Graves K. The Montgomery-Åsberg Depression Scale: reliability and validity. Acta Psychiatrica Scandinavica. 1986;73(5). 10.1111/j.1600-0447.1986.tb02723.x. 10.1111/j.1600-0447.1986.tb02723.x 3751660

[CR13] Depp CA, Mausbach BT, Harmell AL, Savla GN, Bowie CR, Harvey PD, Patterson TL. Meta-analysis of the association between cognitive abilities and everyday functioning in bipolar disorder. Bipolar Disord. 2012;14(3):217–26. 10.1111/J.1399-5618.2012.01011.X. 22548895 10.1111/j.1399-5618.2012.01011.x PMC3396289

[CR14] Dunayevich E, Keck PE, Address †. Prevalence and description of psychotic features in bipolar mania. Curr Psychiatry Rep. 2000;2:286–90. 11122970 10.1007/s11920-000-0069-4

[CR15] Eve Lewandowski K, Balci Camsari G, Lewandowski KE, DePaola J, Camsari GB, Cohen BM, Öngür D. (2009). Tactile, olfactory, and gustatory hallucinations in psychotic disorders: a descriptive study cognition across the psychosis spectrum view project Cognitive Remediation View project Tactile, olfactory, and gustatory hallucinations in psychotic disorders: a descriptive study. *38*(5). 10.47102/annals-acadmedsg.V38N5p383

[CR16] First M, Williams J, Karg R, Spitzer R. Structured clinical interview for DSM-5—Research Version (SCID-5 for DSM-5, Research Version; SCID-5-RV). American Psychiatric Association; 2015.

[CR17] Glahn DC, Bearden CE, Barguil M, Barrett J, Reichenberg A, Bowden CL, Soares JC, Velligan DI. The neurocognitive signature of psychotic bipolar disorder. Biol Psychiatry. 2007;62(8). 10.1016/j.biopsych.2007.02.001. 10.1016/j.biopsych.2007.02.001 17543288

[CR18] Gupta T, DeVylder JE, Auerbach RP, Schiffman J, Mittal VA. Speech illusions and working memory performance in non-clinical psychosis. Schizophr Res. 2018;195:391–5. 10.1016/J.SCHRES.2017.10.031. 29089190 10.1016/j.schres.2017.10.031 PMC5924653

[CR19] Hammersley P, Taylor K, McGovern J, Kinderman P. Attributions for hallucinations in bipolar affective disorder. Behav Cogn Psychother. 2010;38(2):221–6. 10.1017/S1352465809990592. 20047708 10.1017/S1352465809990592

[CR20] Hugdahl K, Nygård M, Falkenberg LE, Kompus K, Westerhausen R, Kroken R, Johnsen E, Løberg EM. Failure of attention focus and cognitive control in schizophrenia patients with auditory verbal hallucinations: evidence from dichotic listening. Schizophr Res. 2013;147(2–3):301–9. 10.1016/J.SCHRES.2013.04.005. 23664588 10.1016/j.schres.2013.04.005

[CR21] Jenkins LM, Bodapati AS, Sharma RP, Rosen C. Working memory predicts presence of auditory verbal hallucinations in schizophrenia and bipolar disorder with psychosis. J Clin Exp Neuropsychol. 2018;40(1). 10.1080/13803395.2017.1321106. 10.1080/13803395.2017.1321106 28562181

[CR22] Kelleher I, Corcoran P, Keeley H, Wigman JTW, Devlin N, Ramsay H, Wasserman C, Carli V, Sarchiapone M, Hoven C, Wasserman D, Cannon M. Psychotic symptoms and population risk for suicide attempt a prospective cohort study. JAMA Psychiatry. 2013;70(9). 10.1001/jamapsychiatry.2013.140. 10.1001/jamapsychiatry.2013.140 23863946

[CR23] Kr Akvik B, Larøi F, Kalhovde AM, Hugdahl K, Kompus K, Salvesen Ø, Stiles TC, As EV-K, Kr B. (2015). *Health and Disability Prevalence of auditory verbal hallucinations in a general population: A group comparison study*. 10.1111/sjop.12236 10.1111/sjop.12236 PMC4744794 26079977

[CR24] Kühn S, Gallinat J. Quantitative Meta-analysis on State and Trait aspects of Auditory Verbal Hallucinations in Schizophrenia. Schizophr Bull. 2012;38(4):779–86. 10.1093/schbul/sbq152. 21177743 10.1093/schbul/sbq152 PMC3406531

[CR25] Kumari R, Chaudhury S, Kumar S. Dimensions of hallucinations and delusions in affective and nonaffective illnesses. Int Sch Res Notices. 2013;2013(1):616304. 10.1155/2013/616304. 10.1155/2013/616304 PMC3755384 23997978

[CR26] Larøi F, Sommer IE, Blom JD, Fernyhough C, Ffytche DH, Hugdahl K, Johns LC, McCarthy-Jones S, Preti A, Raballo A, Slotema CW, Stephane M, Waters F. The characteristic features of auditory verbal hallucinations in clinical and nonclinical groups: state-of-the-art overview and future directions. Schizophr Bull. 2012;38(4):724–33. 10.1093/SCHBUL/SBS061. 22499783 10.1093/schbul/sbs061 PMC3406519

[CR27] Levy B, Medina AM, Weiss RD. Cognitive and psychosocial functioning in bipolar disorder with and without psychosis during early remission from an acute mood episode: a comparative longitudinal study. Compr Psychiatr. 2013;54(6). 10.1016/j.comppsych.2012.12.018. 10.1016/j.comppsych.2012.12.018 PMC4076957 23357126

[CR28] Mancuso SG, Morgan VA, Mitchell PB, Berk M, Young A, Castle DJ. A comparison of schizophrenia, schizoaffective disorder, and bipolar disorder: results from the second Australian national psychosis survey. J Affect Disord. 2015;172:30–7. 10.1016/J.JAD.2014.09.035. 25451392 10.1016/j.jad.2014.09.035

[CR29] Morgan VA, Mitchell PB, Jablensky AV. The epidemiology of bipolar disorder: Sociodemographic, disability and service utilization data from the Australian National Study of Low Prevalence (psychotic) disorders. Bipolar Disord. 2005;7(4). 10.1111/j.1399-5618.2005.00229.x. 10.1111/j.1399-5618.2005.00229.x 16026485

[CR30] Mueser KT, Bellack AS, Brady EU. Hallucinations in schizophrenia. Acta Psychiatrica Scandinavica. 1990;82(1):26–9. 10.1111/j.1600-0447.1990.tb01350.x. 2399817 10.1111/j.1600-0447.1990.tb01350.x

[CR31] Qiu L, Ye J, Ji F, Li G, Li G, Ma X, Li R, Tian H, Wang L, Chen G, Xu Y, Wang W, Jiang D, Pan J, Zhuo C. Common and distinct global functional connectivity density alterations in patients with bipolar disorder with and without auditory verbal hallucination during major depressive episodes. Brain Imaging Behav. 2020;14(6):2724–30. 10.1007/S11682-019-00222-4/FIGURES/2. 31900890 10.1007/s11682-019-00222-4

[CR32] Sajatovic M, Levin J, Fuentes-Casiano E, Cassidy KA, Tatsuoka C, Jenkins JH. Illness experience and reasons for nonadherence among individuals with bipolar disorder who are poorly adherent with medication. Compr Psychiatr. 2011;52(3):280–7. 10.1016/J.COMPPSYCH.2010.07.002. 10.1016/j.comppsych.2010.07.002 PMC3140848 21497222

[CR34] Simonsen C, Sundet K, Vaskinn A, Birkenaes AB, Engh JA, Færden A, Jónsdóttir H, Ringen PA, Opjordsmoen S, Melle I, Friis S, Andreassen OA. Neurocognitive dysfunction in bipolar and schizophrenia spectrum disorders depends on history of psychosis rather than diagnostic group. Schizophr Bull. 2011;37(1). 10.1093/schbul/sbp034. 10.1093/schbul/sbp034 PMC3004191 19443616

[CR35] Smith L, Johns M, L, Mitchell R. Characterizing the experience of auditory verbal hallucinations and accompanying delusions in individuals with a diagnosis of bipolar disorder: a systematic review. Bipolar Disord. 2017;19. 10.1111/bdi.12520. 10.1111/bdi.12520 28804990

[CR37] Strakowski SM, Williams JR, Sax KW, Fleck DE, DelBello MP, Bourne ML. Is impaired outcome following a first manic episode due to mood-incongruent psychosis? J Affect Disord. 2000;61(1–2). 10.1016/S0165-0327(99)00192-5. 10.1016/s0165-0327(99)00192-5 11099745

[CR38] Toh WL, Thomas N, Rossell SL. Auditory verbal hallucinations in bipolar disorder (BD) and major depressive disorder (MDD): a systematic review. J Affect Disord. 2015;184. 10.1016/j.jad.2015.05.040. 10.1016/j.jad.2015.05.040 26066781

[CR39] Tohumcu K, Çuhadar D. The relationship between insight and coping attitudes in bipolar disorder patients. Psychol Health Med. 2024. 10.1080/13548506.2024.2407443. 39329249 10.1080/13548506.2024.2407443

[CR40] Upthegrove R, Chard C, Jones L, Gordon-Smith K, Forty L, Jones I, Craddock N. Adverse childhood events and psychosis in bipolar affective disorder. Br J Psychiatry. 2015;206(3). 10.1192/bjp.bp.114.152611. 10.1192/bjp.bp.114.152611 25614532

[CR41] Varese F, Udachina A, Myin-Germeys I, Oorschot M, Bentall RP. (2011). The relationship between dissociation and auditory verbal hallucinations in the flow of daily life of patients with psychosis. *3*(1), 14–28. 10.1080/17522439.2010.548564

[CR42] Wang TT, Beckstead JW, Yang CY. Social interaction skills and depressive symptoms in people diagnosed with schizophrenia: the mediating role of auditory hallucinations. Int J Ment Health Nurs. 2019;28(6):1318–27. 10.1111/INM.12643. 31433115 10.1111/inm.12643

[CR43] Wang Z, Wang H, Mwansisya TE, Sheng Y, Shan B, Liu Z, Xue Z, Chen X. (2021). The integrity of the white matter in first-episode schizophrenia patients with auditory verbal hallucinations: An atlas-based DTI analysis. *Psychiatry Research - Neuroimaging*, *315*. 10.1016/J.PSCYCHRESNS.2021.111328 10.1016/j.pscychresns.2021.111328 34260985

[CR44] Wels L, Dalkner N, Lenger M, Fellendorf T F., Schönthaler EMD, Harvey P. D., Reininghaus E. Z. Cognitive insight and introspective accuracy in individuals with bipolar disorder: a scoping review. Neurosci Appl. 2023;2:101132. 10.1016/J.NSA.2023.101132. 10.1016/j.nsa.2023.101132 PMC12244013 40655982

[CR45] Wilson S, Sponheim SR. Dimensions underlying psychotic and manic symptomatology: extending normal-range personality traits to schizophrenia and bipolar spectra. Compr Psychiatr. 2014;55(8):1809–19. 10.1016/j.comppsych.2014.07.008 25091283

[CR46] Yalincetin B, Bora E, Binbay T, Ulas H, Akdede BB, Alptekin K. Formal thought disorder in schizophrenia and bipolar disorder: a systematic review and meta-analysis. Schizophr Res. 2017;185:2–8. 10.1016/J.SCHRES.2016.12.015. 28017494 10.1016/j.schres.2016.12.015

[CR47] Young RC, Biggs JT, Ziegler VE, Meyer DA. A rating scale for mania: reliability, validity and sensitivity. Br J Psychiatry. 1978;133(11). 10.1192/bjp.133.5.429. 10.1192/bjp.133.5.429 728692

