# Supplementary material for: Characteristics of people with bipolar disorder I with and without auditory verbal hallucinations
Source: Int J Bipolar Disord. 2025 Feb 14;13:4. doi: 10.1186/s40345-025-00369-8 (PMC11828762; doi:10.1186/s40345-025-00369-8)
Supplement: Supplementary file 1 — Supplementary Material 1 [file 40345_2025_369_MOESM1_ESM.docx]

**Supplementary Table 1:** Demographic Factors and Clinical Scores for Bipolar Disorder Type I (BD-I) Patients with (BP/AVH+) and without (BP/AVH-) Auditory Verbal Hallucinations (AVH) with total Scale for the Assessment for Positive Symptoms (SAPS) score as a covariate (minus AVH score).

| **Parameter** | **BP/AVH+** (N=36) | **BP/AVH-** (N=83) | **Significance** |
| --- | --- | --- | --- |
| Total Years of Education | M=14.29 | M=15.08 | *F*=2.04  *p=*0.16 |
| Subjective SES | M=4.74 | M=5.41 | *F*=1.67  *p=*0.20 |
| YMRS total | M=8.71 | M=5.81 | *F*=2.97  *p=*0.09 |
| MADRS total | M=14.77 | M=13.02 | *F*=.56  *p=*0.46 |

NOTE: Adjusted means are reported

SES: Socioeconomic Status; SAPS: Scale for the Assessment of Positive Symptoms; MADRS: Montgomery Åsberg Depression Rating Scale; YMRS: Young Mania Rating Scale.
